# The effects of alcohol consumption on flow‐mediated dilation in humans: A systematic review

**DOI:** 10.14814/phy2.14872

**Published:** 2021-05-27

**Authors:** Chueh‐Lung Hwang, Mariann R. Piano, Shane A. Phillips

**Affiliations:** ^1^ Department of Physical Therapy College of Applied Health Sciences University of Illinois at Chicago Chicago IL USA; ^2^ School of Nursing Vanderbilt University Nashville TN USA

**Keywords:** alcohol, binge drinking, endothelium, flow‐mediated dilation

## Abstract

Changes in endothelial function may contribute to the positive and negative effects of alcohol consumption on cardiovascular conditions, such as hypertension and coronary artery disease. Numerous studies have used brachial artery flow‐mediated dilation (FMD) to examine the effects of alcohol consumption on endothelial function in humans. However, the findings are inconsistent and may be due to multiple factors such as heterogeneity in subject characteristics, the alcohol use pattern, and amount/dose of alcohol consumed. Therefore, the aim of this systematic review was to investigate the effect of alcohol consumption on brachial artery FMD in humans considering the above‐mentioned factors. This review found that while light to moderate alcohol consumption may have minimal effects on FMD, heavy alcohol consumption was associated with a decrease in FMD. However, most of the published studies included healthy, younger, and male individuals, limiting generalizability to other populations. Future studies should include more women, older subjects, and those from diverse and underrepresented backgrounds.

## Graphical abstract

Numerous studies have used brachial FMD to examine the effects of alcohol consumption on endothelial function, but the findings are inconsistent. This may be due to multiple factors such as heterogeneity in subject characteristics (disease vs. healthy), alcohol type and amount, and measurement timing (acute vs. chronic consumption). Therefore, the purpose of this systematic review was to investigate the effect of alcohol consumption on FMD in human.

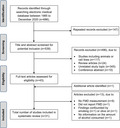

## 1 INTRODUCTION

Over the last several decades there have been many investigations of the effects of alcohol consumption on endothelial function. Endothelial cells play a critical role in maintaining vascular health by releasing endothelial cell‐derived substances such as nitric oxide, a potent vasodilator (Vanhoutte et al., 2016). Impaired endothelial function, characterized by reduced bioavailability of nitric oxide, is an early indicator of blood vessel damage and precursor to atherosclerosis (Bonetti et al., 2003; Choi et al., 2013). Changes in endothelial function may be a mechanism that serves as a contributing mediator of the positive and negative effects of alcohol consumption on cardiovascular conditions, including hypertension and coronary artery disease (Piano, 2017).

Brachial artery flow‐mediated dilation (FMD) is a non‐invasive method used to assess endothelial function in peripheral conduit vessels (Thijssen et al., 2019). The method includes ultrasound imaging of the brachial artery to measure the diameter changes before and after ischemia (typically 5 min) that induces a transient increase in blood flow (i.e., hyperemia) and thus shear stress. The increase in shear stress stimulates the endothelium to release nitric oxide, which diffuses into vascular smooth muscle cells, causing vasodilation. Therefore, brachial artery FMD is considered a measure of endothelium‐ and nitric oxide‐dependent vasodilation. In experimental and clinical studies, FMD has been extensively used as a surrogate marker of future cardiovascular events (Inaba et al., 2010; Matsuzawa et al., 2015; Ras et al., 2013; Xu et al., 2014). Several meta‐analyses have reported a significant association between a lower value of brachial artery FMD and a higher risk of future cardiovascular events (Inaba et al., 2010; Matsuzawa et al., 2015; Ras et al., 2013; Xu et al., 2014).

Numerous studies have used brachial FMD to examine the effects of alcohol consumption on endothelial function, however findings among the studies are inconsistent, making it difficult to formulate conclusions about the effects of alcohol consumption on brachial artery FMD. Therefore, this systematic review aims to provide a summary of brachial FMD studies conducted in human beings with the consideration of variables such as subject characteristics (sex, age, race, or disease status), alcohol type (wine, beer, or liquor), alcohol amount (how many drinks or grams of alcohol/day), and acute versus short‐ and long‐term alcohol consumption. Overall, this systematic review will provide a better understanding of the benefit or harm of alcohol consumption on brachial FMD and provide directions for future research.

## 2 METHODS

### 2.1 Search and selection criteria

A systematic literature search was conducted using electronic medical databases (i.e., PubMed, CINHAL and EMBASE, February 13, 2021). The search was conducted independently by a medical librarian in accordance with PRISMA guidelines and in consultation with authors C.‐L.H. and M.R.P. using Mesh terms related to alcohol use and endothelial function (see Table S1 for full search strategies).

Studies were eligible for inclusion in this systematic review if the following inclusion criteria were met: (1) studies conducted in human adult (age ≥18 years) subjects following: an acute (one time), short‐term (several weeks), or long‐term (repeated, at least a year) period of alcohol consumption, (2) studies reported the amount of alcohol consumed and duration of consumption, and (3) studies used FMD measurements of the brachial artery with ultrasound and presented the FMD response to hyperemia either in absolute (mm) or relative (%) change. The screening process included the initial review of titles and abstracts and the full‐text review was performed independently by authors C.‐L.H. and M.R.P. Articles were excluded if the article did not meet the inclusion criteria. Articles included in the final review were done so by consensus, with final inclusion decisions being made by author S.A.P.

### 2.2 Data extraction

Primary outcomes of the included studies were alcohol consumption and brachial artery FMD. Depending on study design and purpose, FMD data were extracted at the following time points: (1) at baseline, (2) following acute alcohol consumption, (3) following short‐term alcohol consumption, and/or (4) following a history of long‐term, repeated alcohol consumption. For studies investigating the acute effect of alcohol, we noted the timing of the FMD measurement after drinking. In order to evaluate the contribution of endothelial function to FMD, endothelium‐independent vasodilation data (if tested) were noted from the included studies. We recorded, alcohol drink type, amount (alcohol concentration or how many drinks), and drinking duration/history length. When calculating the alcohol content in grams for studies which did not specify alcohol content, we estimated that the alcohol content was 13% v/v for red wine with an alcohol density of 0.785 g/ml. For studies conducted in the European Union, unless otherwise noted, we assumed one standard drink contained 12 g of alcohol. If the amount of alcohol consumption was normalized to body weight, we assumed a body weight of 75 kg for an adult subject. Subject characteristics were assessed by extracting health status and other quantitative characteristics such as age, sex, race, body mass index and systolic blood pressure. Data were extracted independently by C.‐L.H and M.R.P and then confirmed by S.A.P.

### 2.3 Study quality assessment

To evaluate the quality of the included studies, we used the National Institute for Health and Clinical Excellence (NICE) quality assessment tool. This tool consists of the following eight questions: (1) Was the case series collected in more than one center (i.e., multicenter study)? (2) Is the hypothesis, aim, or objective of the study clearly described? (3) Are the inclusion and exclusion criteria (case definition) clearly reported? (4) Is there a clear definition of the outcomes reported? (5) Were data collected prospectively? (6) Is there an explicit statement that patients were recruited consecutively? (7) Are the main findings of the study clearly described? (8) Are outcomes stratified (e.g., by abnormal results, disease stage, patient characteristics)?

To evaluate the quality of brachial artery FMD measurements, we used the 2019 guideline published by Thijssen et al. (2019). Briefly, the 2019 guideline recommends the following: (1) for subject preparation: subjects should fast for ≥6 h and avoid exercise for ≥24 h and caffeine or alcohol for ≥12 h. Medication use, including the timing of the medication should be considered and subjects should rest in a supine position, in a quiet and darkened room for 10–15 min before FMD measurement. FMD measurements should be performed at a standardized time of day to prevent diurnal variation and for female subjects, should be performed at a standardized phase of menstrual cycle to prevent hormone variation; (2) for the FMD protocol: FMD should be assessed by measuring diameter of the brachial artery at baseline for >30 s and after hyperemia for ≥3 min. Hyperemia is induced by inflating the cuff (distal to the brachial arteries) for 5 min and then rapidly deflating it. The cuff pressure should be at least 50 mmHg above systolic blood pressure. To test endothelium‐independent vasodilation, brachial artery maximum diameter changes are measured after the administration of sublingual glyceryl trinitrate (the dose should be reported); and (3) for techniques: blood velocity and diameter of the brachial artery should be recorded continuously using a simultaneous live duplex ultrasound with a linear probe of ≥7.5 MHz and an intonation of ≤60–70° (Table S2). For analyzing brachial artery diameter, a continuous edge detection and wall tracking software should be used with automated mathematical algorithms to calculate the peak value. Investigators need to report the baseline diameter and FMD response in absolute (in mm) and relative change (in %).

## 3 RESULTS

### 3.1 Study selection

**FIGURE 1:**
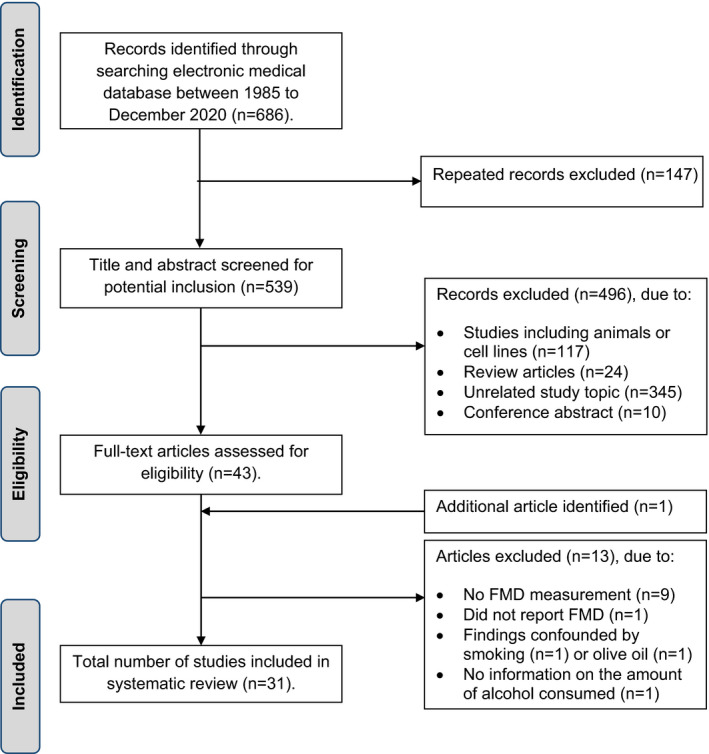
Study selection flow

The search produced 155 records from Pubmed, 75 records from CINHAL, and 456 records from EMBASE. After cross‐checking the repeated records, a total of 539 articles were reviewed for possible inclusion (Figure 1). After study titles and abstracts were reviewed, 43 full‐text articles were selected for full‐text review. During the review process, one article (Djousse et al., 1999) was identified from the references in one of the reviewed articles (Zilkens et al., 2005). Nine articles used other vascular measures or vascular beds (Bian et al., 2018; Botden et al., 2011; Cioni et al., 2013; Ferguson et al., 2008; Flesch et al., 1998; Kiviniemi et al., 2010; Tawakol et al., 2004; Tousoulis et al., 2008; Vauzour et al., 2010) and one did not report FMD in response to hyperemia either in absolute or relative change (Guarda et al., 2005). Two studies had findings confounded by either smoking (Papamichael et al., 2004) or olive oil consumption (Karatzi et al., 2008), and one study only reported alcohol use over the past 30 days (yes/no) with no information on the amount of alcohol consumed (Hill et al., 2020). For these reasons, we excluded these 13 studies, and therefore, 31 studies met inclusion criteria and were included in the research synthesis (Agewall et al., 2000; Andrade et al., 2009; Bau et al., 2005; Boban et al., 2006; van Bussel et al., 2018; Coimbra et al., 2005; Cuevas et al., 2000; Djousse et al., 1999; Di Gennaro et al., 2007, 2012; Goslawski et al., 2013; Hampton et al., 2010; Hashimoto et al., 2001; Hijmering et al., 2007; Huang et al., 2010; Karatzi et al., 2004, 2013; Luo et al., 2017; Maiorano et al., 1999; Muggeridge et al., 2019; Oda et al., 2017, 2020; Schaller et al., 2010; Spaak et al., 2008; Suzuki et al., 2009; Tanaka et al., 2016; Teragawa et al., 2002; Vlachopoulos et al., 2003; Whelan et al., 2004; Zilkens et al., 2003, 2005). Among these studies, only six studies were published within the past 5 years.

### 3.2 Study characteristics

#### 3.2.1 Studies examining the acute effects of alcohol

Fourteen studies investigated the acute effects of alcohol (one‐time alcohol consumption) on FMD (Table 1). Among these studies, various research designs were used: 10 studies used a randomized, crossover design (Agewall et al., 2000; Boban et al., 2006; Hampton et al., 2010; Hashimoto et al., 2001; Karatzi et al., 2004, 2013; Muggeridge et al., 2019; Spaak et al., 2008; Vlachopoulos et al., 2003; Whelan et al., 2004), two used a randomized, parallel design (Bau et al., 2005; Hijmering et al., 2007), and two used a crossover design (Djousse et al., 1999; Schaller et al., 2010). Eleven out of the 14 studies enrolled healthy adults (study sample size, *n*, ranging from 7 to 100; total *n* = 224), while the other three studies enrolled patients with coronary artery disease (*n* = 29; Karatzi et al., 2004; Whelan et al., 2004) or type 2 diabetes (*n* = 12; Schaller et al., 2010). Except for one study (Schaller et al., 2010), all other acute studies included a non‐alcoholic or low‐alcoholic beverage group. Most of the studies had predominantly male and young participants (mean age ranged between 21 and 36 years). With regard to race/ethnicity, only one study was conducted in Asia (Hashimoto et al., 2001), while the remaining 12 studies were conducted in non‐Asian countries. The majority of studies conducted in non‐Asian countries did not provide race/ethnicity data: one study noted the inclusion of only non‐Asian participants (Spaak et al., 2008) and one study noted the inclusion of “predominantly Caucasian participants” (Djousse et al., 1999).

**TABLE 1:** Acute effects of alcohol on FMD (*N* = 14)

Study	Study design	Subject characteristics					One‐time alcohol consumption				Outcomes	
		*n* (F)	Age (years)	BMI (kg/m^2^)	SBP (mmHg)	Baseline FMD (%)	With meal?	Type	Alcohol content	Drinking volume and duration	Timing after alcohol consumption	FMD after alcohol consumption versus baseline and % change
*Healthy adults (N = 11)*												
Agewall et al. (2000)	Randomized, crossover	12 (4)	31 ± 4	25 ± 3	121 ± 6	3.9 ± 2.5	No	Red wine	12.5% v/v	250 ml over 10 min	30–60 min	↔
							No	De‐alcoholized red wine	<0.5% v/v	250 ml over 10 min	30–60 min	↑43%*
Bau et al. (2005)	Randomized, parallel	50 (0)	21 ± 2	23 ± 2	NR^a^	5.1 ± 4.2	No	Water, citric acid, and glucose	0 g	500 ml over 30 min	4 h 13 h^b^	↔ ↔
		50 (0)			NR^a^	4.2 ± 4.2	No	Alcohol + water, citric acid, and glucose	60 g	500 ml over 30 min	4 h 13 h^b^	↓42%* ↔
Boban et al. (2006)	Randomized, crossover	9 (0)	25–40	25 ± 12	NR^a^	8.5 ± 1.8	No	Water	0 g	3 ml/kg bw	60 min	↔
						6.5 ± 1.8	No	Dealcoholized red wine	0.21% v/v	3 ml/kg bw	60 min	↔
						7.3 ± 4.2	No	Ethanol water solution	14.0% v/v	3 ml/kg bw	60 min	↔
						8.8 ± 2.7	No	Polyphenols‐stripped red wine	13.3% v/v	3 ml/kg bw	60 min	↔
						7.1 ± 1.8	No	Red wine	14.0% v/v	3 ml/kg bw	60 min	↔
Djousse et al. (1999)	NR	13 (6)	32 ± 9	25 ± 3	121 ± 12	NR^a^	Yes	Isocaloric amount of Coca‐Cola Classic	0 g	NR	2 h 4 h 6 h	↔ ↔ ↔
						NR^a^	Yes	3 ml/kg of red wine	NR	NR	2 h 4 h 6 h	↔ ↔ ↔
Hampton et al. (2010)	Randomized, three‐way, cross‐over	10 (5)	22 ± 4	24 ± 3	NR	5.7 ± 1.2	Yes	Water	0 g	175 ml; time NR	30 min 60 min	↔
							Yes	Organic red grape juice + water	0 g	123 ml juice + 53 ml water; time NR	30 min 60 min	Overall ↑*
							Yes	Organic red grape juice + vodka	21 g	175 ml; time NR	30 min 60 min	Overall ↑*
Hashimoto et al. (2001)	Randomized, cross‐over	11 (0)	34 ± 3	NR	110 ± 13	NR^a^	No	Water	0 g	500 ml over 30 min	30 min 120 min	↔ ↔
					112 ± 13	NR^a^	No	Dealcoholized red wine	0 g	500 ml over 30 min	30 min 120 min	↑ ↑
					116 ± 17	NR^a^	No	Red wine	0.8 g/kg bw	500 ml over 30 min	30 min 120 min	↔ ↑
					112 ± 13	NR^a^	No	Japanese vodka	0.8 g/kg bw	500 ml over 30 min	30 min 120 min	↓ ↓
Hijmering et al. (2007)	Randomized	10 (3)	35 ± 11	≤30	≤140	7.3 ± 4.8	No	Barcardi Breezer (low polyphenolic)	33 g	275 ml over 45 min	45 min	↓62%
							No	Barcardi Breezer (low polyphenolic)	66 g	Two times of 275 ml over 45 min interspersed by 45 min	45 min	↓84%
		10 (4)	36 ± 9	≤30	≤140	8.6 ± 1.8	No	Red wine (high polyphenolic)	34 g	110 ml over 45 min	45 min	↓79%
							No	Red wine (high polyphenolic)	68 g	Two times of 110 ml over 45 min interspersed by 45 min	45 min	↓86%
Karatzi et al. (2013)	Randomized, single‐blind, cross‐over	17 (0)	28 ± 5	24 ± 3	115 ± 6	4.7 ± 3.2	Yes	Dealcoholized beer	0 g	800 ml over 15 min	1 h 2 h	Overall↔
						2.5 ± 2.0	Yes	Beer and water	~20 g	800 ml over 15 min	1 h 2 h	Overall↔ (but higher than other 2 groups)*
						3.9 ± 2.2	Yes	Vodka and water	~20 g	800 ml over 15 min	1 h 2 h	Overall↔
Muggeridge et al. (2019)	Randomized, counterbalanced, crossover	7 (6)	57 ± 3	31 ± 5	127 ± 14	6.4 ± 2.9	Yes	Water	0	250 ml	2 h	↓29%
						6.7 ± 2.8	Yes	Orange juice	0	250 ml	2 h	↓43%
						6.1 ± 3.8	Yes	Green tea	0	250 ml	2 h	↓29%
						6.8 ± 4.2	Yes	Red wine	NR	250 ml	2 h	↓23%
Spaak et al. (2008)	Randomized, single‐blind, water‐controlled	13 (6)	35 ± NR	23 ± NR	111 ± 13	6.5 ± 7.2	No	Water	0	Equal volume to red wine over 5 min	10 min	↔
							No	Water	0	Equal volume to red wine over the same time period min	10 min	↔
					112 ± 14	6.9 ± 4.3	No	95% ethanol + water	Equal amount to red wine	First drinks: equal volume to red wine over 5 min	10 min	↔
							No	95% ethanol + water	Equal amount to red wine	Following the first drinks and BAC dropping to 25–30 mg/dl, second drink: equal volume to red wine over 5 min	10 min	↔
					109 ± 15	6.7 ± 4.0	No	Red wine	12%	First drinks: Volume bringing BAC to 40 mg/dl over 5 min	10 min	↔
							No	Red wine	12%	Following the first drinks and BAC dropping to 25–30 mg/dl, second drink: volume bringing BAC to ~90 mg/dl over 5 min	10 min	↔ (but lower than water group)*
Vlachopoulos et al. (2003)	Randomized, sham controlled, single‐blind, crossover	12 (5)	32 ± 3	NR	112 ± 3	5.8 ± 1.2	No	Grapefruit juice	0	200 ml over 10 min	30 min	↔
					117 ± 3	6.1 ± 1.1	No	Pure alcohol + grapefruit juice	1 oz	~230 ml over 10 min	30 min	↔
*Coronary artery disease (N = 2)*												
Karatzi et al. (2004)	Randomized, double‐blind, cross‐over	15 (0)	53 ± 10	28 ± 2	118 ± 14	2.4 ± 2.3	Yes	De‐alcoholized red wine	<1%	250 ml over 15 min	30 min 60 min 90 min	↔ ↔ ↔
					115 ± 12	3.8 ± 2.4	Yes	Red wine	12%	250 ml over 15 min	30 min 60 min 90 min	↔ ↓61%* ↔
Whelan et al. (2004)	Randomized, single‐blind, cross‐over	14 (0)	58 ± 6	28 ± 4	135 ± 14	Figure	Yes	Isocaloric cordial	0	4 ml/kg bw over 30 min	1 h 6 h	↔ ↔
					135 ± 10	1.8 ± 1.7	Yes	Red wine	13.5% v/v	4 ml/kg bw over 30 min	1 h 6 h	↔ ↑194%
					135 ± 13	1.6 ± 1.9	Yes	White wine	13% v/v	4 ml/kg bw over 30 min	1 h 6 h	↔ ↑89%
*Type 2 diabetes (N = 1)*												
Schaller et al. (2009)	Cross‐over	12 (0)	64 ± 6	28 ± 6	143 ± 7	5.1 ± 0.9	Yes^c^	None	—	—	3 h	↔
					151 ± 24	5.1 ± 0.9	Yes^c^	40% Vodka	40 g	168 ml over 15 min	3 h	↑54%

Note

Data are *n* or mean ± SD.

Abbreviations: BMI, body mass index; bw, body weight; FMD, flow‐mediated dilation; *N*, number of the reviewed studies; *n*, number of the study participants; NR, not reported; SBP, systolic blood pressure.

^a^ Data were only presented in a figure.

^b^ Standard meals and snacks were provided at least 9 h prior to the FMD measurements.

^c^ Insulin‐modified frequently sampled intravenous glucose tolerance test.

* Significant between‐group differences.

#### 3.2.2 Studies examining the short‐term effects of alcohol

Of the six studies investigating the short‐term effects of alcohol (several weeks) on FMD (Table 2), four studies used a randomized design, with a non‐alcohol control group (Coimbra et al., 2005; Huang et al., 2010; Zilkens et al., 2005) or a low‐alcohol experimental group (Zilkens et al., 2003). Among these studies, the sample sizes ranged from 5 to 24 per group (total *n* = 173), and subjects had no history of cardiovascular disease and were predominantly male and young and middle‐aged. With regard to race/ethnicity, two studies included only White participants (Zilkens et al., 2003, 2005), and one was conducted in Asia (Huang et al., 2010), while three other studies conducted in Chile, Brazil and Australia did not report race/ethnicity.

**TABLE 2:** Short‐term effects of alcohol on FMD (*N* = 6)

Study	Study design	Subject characteristics						Alcohol intervention			Outcomes FMD after intervention versus baseline
		Health status	*n* (F)	Age (years)	BMI (kg/m^2^)	SBP (mmHg)	Baseline FMD (%)	Type	Volume (alcohol content)	Length	
Andrade et al. (2009)	Case‐controlled, non‐randomized	Healthy controls	7 (2)	37 ± 7	25 ± 3	134 ± 6	13.1 ± 1.7	Red wine	250 ml/day after an evening meal	15 days	↔
		Arterial hypertension (>140/90 mmHg)	9 (2)	45 ± 7	28 ± 2	153 ± 8	4.7 ± 1.5	Red wine	250 ml/day after an evening meal		↔
		Hypercholesterolemia (LDL‐c >160 mg/dl)	10 (3)	43 ± 7	26 ± 3	141 ± 15	6.9 ± 12.3	Red wine	250 ml/day after an evening meal		↑113%
Coimbra et al. (2005)	Randomized, cross‐over	Hypercholesterolemia	16 (8)	52 ± 8	25 ± 2	122 ± 10	10.9 ± 7.4	Grape juice	500 ml/day	14 days	↑55%
					25 ± 2	123 ± 10	10.1 ± 6.4	Red wine	250 ml/day		↑54%
Cuevas et al. (2000)	Non‐randomized, non‐cross‐over	Healthy normotensive receiving high‐fat diet	5 (0)	20–28	20–25	NR	−2.9 ± 2.1	Red wine	240 ml/day	30 days	↑326%
	Non‐randomized, non‐cross‐over	Healthy normotensive receiving control diet	6 (0)	20–28	20–25	NR	3.1 ± 3.9	Red wine	240 ml/day	30 days	↔
Huang et al. (2010)	Randomized, diet‐controlled	Healthy	20 (3)	34 ± 4	24 ± 4	120 ± 9	NR	Pure water	100 ml/day	3 weeks	↔
			20 (6)	33 ± 4	25 ± 4	121 ± 9	7.4 ± 2.7	Red wine	100 ml/day (12.5%)		↑35%
			20 (5)	34 ± 4	25 ± 4	121 ± 9	NR	Beer	250 ml/day (5%)		↔
			20 (3)	33 ± 4	24 ± 4	125 ± 9	NR	Vodka	30 ml/day (37.5%)		↔
Zilkens et al. (2003)	Randomized, cross‐over	Healthy normotensive	16 (0)	51 ± NR	27 ± 4	125 ± 16	6.8 ± 2.6	Low‐alcohol content beer	7.9 ± 1.6 g of alcohol/day	4 weeks	↔
								Regular alcohol consumption (92% from beer)	72.4 ± 5.0 g of alcohol/day		↔
Zilkens et al. (2005)	Randomized, cross‐over	Healthy normotensive	24 (0)	53 ± 8	25 ± 3	125 ± 2^a^	NR	No alcohol or grape products	0 ml/day (0%)	4 weeks	6.1 ± 2.1
						124 ± 2^a^		Dealcoholized red wine	375 ml/day (0%)		5.7 ± 2.5
						125 ± 2^a^		Beer	1,125 ml/day (4.6% v/v)		6.4 ± 3.2
						123 ± 2^a^		Red wine	375 ml/day (13% v/v)		6.5 ± 3.1

Note

Data are *n* or mean ± SD.

Abbreviations: BMI, body mass index; FMD, flow‐mediated dilation; *N*, number of the reviewed studies; *n*, number of the study participants; NR, not reported; SBP, systolic blood pressure.

^a^ Based on the 24‐h ambulatory data.

#### 3.2.3 Studies examining the effect of long‐term alcohol consumption

Eleven studies examined the effects of repeated, long‐term (at least 1 year) alcohol consumption on FMD, using a cross‐sectional design and a control or abstinent group (Table 3). Six out of the 11 studies had sample sizes ranging from 36 to 108, while the other five studies were population‐based with the sample sizes ranging from 404 to 2734 (Table 3). Except for two studies (Oda et al., 2020; Suzuki et al., 2009), most studies had predominantly male participants or included only male participants. The mean age across these studies ranged between 23 and 71 years. Five out of the 11 studies were conducted in Asian countries (Luo et al., 2017; Oda et al., 2017, 2020; Tanaka et al., 2016; Teragawa et al., 2002). Among studies conducted in non‐Asian countries, one reported that nearly half of participants were Caucasians (Goslawski et al., 2013), one study reported that ~17% participants were non‐Hispanic Black and ~15% were non‐Hispanic White (Suzuki et al., 2009), while the other studies did not specify race/ethnicity.

**TABLE 3:** Long‐term effect of alcohol consumption on FMD (*N* = 11)

Study	Subject characteristics						Alcohol consumption		Outcomes FMD versus reference group
	Alcohol use group	*n* (F)	Age (years)	BMI (kg/m^2^)	SBP (mmHg)	FMD (%)	History length	Amount	
*Sample‐based cross‐sectional studies*									
Di Gennaro et al. (2007)	Abstainers	39 (12)	48 ± 10	24 ± 0	118 ± 11	14.9 ± 7.4	Lifetime	Never	Reference group
	Patients with history of chronic alcoholism following alcohol abstinence	42 (10)	48 ± 10	25 ± 0	128 ± 13	10.1 ± 4.6	6–41 years of alcohol use, followed by 6–144 months of abstinence	8–32 drinks/day; abstinence	↓32%
Di Gennaro et al. (2012)	Teetotalers	35 (8)	46 ± 11	24 ± 0	118 ± 10	14.8 ± 7.5	Lifetime	<10 drinks	Reference group
	Heavy alcoholics	29 (2)	45 ± 9	25 ± 4	130 ± 12	10.6 ± 6.2	2–42 years of alcohol use, followed by 6–14 months of abstinence	86–215 g/day of alcohol; abstinence	↓28%
					145 ± 20	8.5 ± 5.4	2–42 years	86–215 g/day of alcohol	↓43% (↓19% vs. abstinence group)
Goslawski et al. (2013)	Abstainers	19 (9)	25 ± NR	24 ± 1	114 ± 3	11.0 ± 3.2	Past year	≤5 drinks	Reference group
	Repeated binge drinkers^a^	17 (6)	23 ± NR	25 ± 1	120 ± 8	8.4 ± 2.9	4 ± 2 years	6 ± 4 binge‐drinking episode over the past month	↓24%
Luo et al. (2017)	Abstainers	30 (0)	55 ± 8	26 ± 2	129 ± 6	13.4 ± 9.4	Lifetime	Never	Reference group
	Alcoholics with mild alcohol use	30 (0)	56 ± 7	25 ± 3	120 ± 11	12.8 ± 0.4	5–8 years	≤90 mg/day of alcohol (2–3 beers), 3–5 days/week	↔
	Alcoholics with moderate alcohol use	31 (0)	58 ± 6	26 ± 1	125 ± 5	7.6 ± 0.2	9–20 years	>90 and <150 mg/day of alcohol, 3–5 days/week	↓43% (↓41% vs. mild group)
	Alcoholics with severe alcohol use	31 (0)	54 ± 7	26 ± 2	131 ± 6	5.9 ± 0.2	>10 years	≥150 mg/day of alcohol (>4 beers), 6–7 days/week	↓56% (↓54% vs. mild group and ↓22% vs. moderate group)
Mairano et al. (1999)	Healthy controls	20 (0)	31 ± 5	27 ± NR	124 ± 6	13.7 ± 4.7	NR	NR	Reference group
	Alcoholics	20 (0)	32 ± 5	27 ± NR	127 ± 7	6.3 ± 3.7	At least 8 years of alcohol use, followed by 3‐month abstinence	75 g/day of alcohol; abstinence	↓54%
Teragawa et al. (2002)	Abstainers with CAD	54 (0)	64 ± 14	24 ± 3	108 ± 15	2.3 ± 1.5	≥1 year	0 drinks/week	Reference group
	Drinkers with CAD	54 (0)	66 ± 7	24 ± 2	109 ± 15	3.8 ± 1.5	NR	≥1 drink/week	↑65%
*Population‐based cohort cross‐sectional studies*									
Oda et al. (2017)	General population (Asian)	733 (0)	48 ± 8	24 ± 3	127 ± 16	6.6 ± 3.0	Past year	0 g/week	Reference group
		1168 (0)	47 ± 9	23 ± 3	127 ± 15	6.2 ± 3.0	Past year	>0 to 140 g/week	↓ OR 1.38 (95%CI 1.10–1.75)
		405 (0)	49 ± 8	24 ± 3	129 ± 16	6.0 ± 3.0	Past year	>140 to 280 g/week	↓ OR 1.36 (95%CI 1.01–1.82)
		225 (0)	49 ± 8	24 ± 3	130 ± 17	5.5 ± 2.9	Past year	>280 to 420 g/week	↓ OR 2.05 (95%CI 1.46–2.87)
		203 (0)	49 ± 7	24 ± 3	129 ± 17	5.3 ± 3.0	Past year	>420 g/week	↓ OR 2.04 (95%CI 1.43–2.89)
Oda et al. (2020)	General population (Asian)	390 (390)	47 ± 14	22 ± 4	121 ± 8	7.3 ± 3.8	Past year	0 g/week	Reference group
		240 (240)	43 ± 13	21 ± 3	115 ± 17	7.2 ± 3.5	Past year	>0 to 140 g/week	↔
		50 (50)	39 ± 13	20 ± 2	108 ± 14	8.2 ± 4.3	Past year	>140 to 280 g/week	↔
		22 (22)	42 ± 17	21 ± 3	117 ± 24	5.9 ± 2.5	Past year	>280 g/week	↓34% when matched for age and medical histories
Suzuki et al. (2009)	General population, stroke‐free	884 (500)	67 ± 9	28 ± 5	143 ± 19	5.5 ± 3.8	Lifetime	<1 drink/month	Reference group
							Lifetime	1 drink/month to 2 drinks/day	↑ OR 1.69 (95%CI 1.17–2.44)
							Lifetime	>2 drinks/day	↔ OR 1.56 (95%CI 0.96–2.54)
Tanaka et al. (2016)	General population (Asian)	81 (0)	53 ± NR	25 ± NR	123 ± NR	7.2 ± NR	Lifetime	Never	Reference group
		33 (0)	60 ± NR	25 ± NR	124 ± NR	6.4 ± NR	Lifetime	Former	↔ OR 1.76 (95%CI 0.69–4.50)
		114 (0)	55 ± NR	24 ± NR	125 ± NR	7.5 ± NR	Lifetime	<23 g/day	↔ OR 0.86 (95%CI 0.42–1.76)
		74 (0)	56 ± NR	24 ± NR	129 ± NR	6.7 ± NR	Lifetime	23–46 g/day	↔ OR 0.98 (95%CI 0.45–2.12)
		102 (0)	55 ± NR	24 ± NR	132 ± NR	5.7 ± NR	Lifetime	≥46 g/day	↓ OR 2.39 (95%CI 1.15–4.95)
van Bussel et al. (2017)	Hoorn study	139 (105)	71 ± 7	28 ± 5	142 ± 22	0.14 ± 0.11 mm	Past year	No consumption of alcohol‐containing beverages	Reference group
		414 (177)	69 ± 7	28 ± 4	142 ± 20	0.16 ± 0.17 mm	Past year	Men: >0 and ≤20 g/day women: >0 and ≤10 g/day	↔ β −0.03 (95%CI −0.25 to 0.19)
		248 (117)	67 ± 7	28 ± 4	142 ± 20	0.20 ± 0.18 mm	Past year	Men: >20 g/day women: >10 g/day	↔ β 0.17 (95%CI −0.07 to 0.41)

Note

Data are *n* or mean ± SD.

Abbreviations: BMI, body mass index; FMD, flow‐mediated dilation; *N*, number of the reviewed studies; *n*, number of the study participants; NR, not reported; SBP, systolic blood pressure.

^a^ Binge drinking was defined as ≥5 drinks for men and ≥4 drinks for women over 2 h.

### 3.3 Alcohol consumption

#### 3.3.1 Studies examining the acute effects of alcohol

In nearly half of the acute studies (numbers of the studies, *N*; *N* = 6), the alcohol consumption protocol included consuming the alcoholic beverage with a meal (Table 1), while in one study, subjects did not receive a meal, but received intravenous glucose as part of a glucose tolerance test (Schaller et al., 2010). Red wine was the most frequently administered type of alcoholic beverage (*N* = 9), followed by vodka (*N* = 4) or pure alcohol (*N* = 4) combined with other ingredients, such as water or juice. De‐alcoholized red wine (*N* = 5), pure water (*N* = 5), and juice (*N* = 3) were often served as the non‐alcoholic or low‐alcoholic beverage to the control group. Overall, alcohol content ranged from 20 to 68 g, drinking volume ranged from 110 to 800 ml, and drinking duration ranged from 5 to 45 min. Two studies testing red wine consumption did not report alcohol content (vol% or grams of alcohol/drink; Djousse et al., 1999; Muggeridge et al., 2019); four studies individualized alcohol content/drinking volume to body weight (Boban et al., 2006; Hashimoto et al., 2001; Whelan et al., 2004) or blood alcohol concentration response (Spaak et al., 2008).

#### 3.3.2 Studies examining the short‐term effects of alcohol

In this category of studies, five out of the six studies included the consumption of a designated amount of red wine as the alcoholic beverage (Table 2) and in two of these studies, other types of alcoholic beverage (such as beer or vodka) were also included (Huang et al., 2010; Zilkens et al., 2005). In most of the studies, the alcohol content per day ranged from 10 to 41 g/day (qualified as moderate alcohol consumption, 2–3 standard drinks/day). In the study by Huang et al., alcohol content is qualified as mild alcohol consumption (10 g/day of alcohol; ~1 drink/day; Huang et al., 2010) and another study by Zilkens et al. included an intervention on reducing alcohol intake from ~5 drinks/day (heavy alcohol consumption) to ~1 drink/day (Zilkens et al., 2003). The duration of the alcohol consumption intervention lasted from 2 to 4 weeks.

#### 3.3.3 Studies examining the effect of long‐term alcohol consumption

In studies examining the effects of repeated, long‐term alcohol consumption, investigators primarily used self‐report questionnaires to determine alcohol consumption levels and patterns and all subjects had a history of consuming alcohol for more than a year (Table 3). All studies noted the amount of alcohol consumed in g/day of alcohol, ranging from 0 to >60 g/day of alcohol. Four of the cross‐sectional studies enrolled patients with a history of alcoholism or history of alcohol use ranging from 26 to 384 g/day of alcohol. Among these studies, the duration of alcohol use was variable and ranged from 2 to 42 years (Di Gennaro et al., 2007, 2012; Luo et al., 2017; Maiorano et al., 1999). In three of the four studies, FMD was also measured in subjects/patients who abstained from alcohol. Similar to the duration of alcohol use, the period of abstention was variable and ranged from 6 to 144 months (Di Gennaro et al., 2007, 2012; Maiorano et al., 1999). One study included participants with an average of a 4‐year duration of repeated binge drinking behavior and binge drinking was defined as at least one binge drinking episode over the past 2 weeks (Goslawski et al., 2013).

### 3.4 Quality of the study and FMD measurements

Among all the studies (Tables 1, 2, 3), 12 studies enrolled subjects from Europe, seven from Asia, four from Oceania, four from North America and four from South America. Only three studies were multi‐centered (Table S2). All studies described the study hypothesis/aim/objective. Some studies did not clearly report the inclusion and exclusion criteria (Agewall et al., 2000; Bau et al., 2005; Boban et al., 2006; Djousse et al., 1999; Hashimoto et al., 2001; Karatzi et al., 2004; Spaak et al., 2008; Vlachopoulos et al., 2003) and one study did not report a clear definition of the outcomes (Hampton et al., 2010). The majority of the studies prospectively collected data. Only two studies had an explicit statement that patients were recruited consecutively (Maiorano et al., 1999; Teragawa et al., 2002). All studies clearly described the main findings, including brachial artery FMD. Cross‐sectional and cohort studies stratified FMD by the history of repeated alcohol use (Table 3), and one study stratified FMD response to alcohol use by disease status (Andrade et al., 2009). Other studies did not stratify findings (Table S2).

None of the studies fully met the 2019 guideline for subject preparation for FMD measurements (Table S3; Thijssen et al., 2019). Except for three studies (Hampton et al., 2010; Muggeridge et al., 2019; Oda et al., 2020), studies enrolling female subjects did not consider the phase of menstrual cycle. Most of the studies did not provide sufficient information about the FMD measurement protocol (such as duration for baseline recording), of which two studies did not report any details (Hijmering et al., 2007; Karatzi et al., 2013). Among the included studies, 18 studies reported endothelium‐independent vasodilation. Most of the studies did not describe the analysis process in detail and did not present blood flow/velocity and/or FMD response in absolute change.

### 3.5 Synthesis of study findings

#### 3.5.1 Acute effects of alcohol on FMD

As noted in Table 1, in healthy adults, FMD responses were measured at different time points ranging from 30 min to several hours (4–13 h) after alcohol consumption and findings are variable. Among studies examining alcohol content <30 g (~2 drinks), five studies found no effect of alcohol on FMD (Boban et al., 2006; Djousse et al., 1999; Hampton et al., 2010; Muggeridge et al., 2019; Vlachopoulos et al., 2003). Karatzi et al. reported no change in FMD following vodka (20 g of alcohol), but the consumption of beer (20 g of alcohol) was associated with a small but significant increase in overall FMD during the 2‐h period following the beer consumption (Karatzi et al., 2013). Agewall et al. found that 250 ml of de‐alcoholized red wine increased FMD 30–60 min after the consumption, while the same amount of red wine with alcohol (~25 g of alcohol) did not change FMD (Agewall et al., 2000). One study compared different amounts/concentrations of red wine (Spaak et al., 2008) and found the following: (1) FMD did not change after the consumption of red wine with drinking volume bringing the blood alcohol level to 40 mg/dl, and (2) there was an overall decrease in FMD with higher drinking volume which brought the blood alcohol level to ~90 mg/dl (Spaak et al., 2008). Three studies with the consumed amount of alcohol ranging 33–68 g found that alcohol acutely decreased FMD 30 min to 4 h following the consumption (Bau et al., 2005; Hashimoto et al., 2001; Hijmering et al., 2007).

Only four out of the 11 studies in healthy subjects evaluated endothelial‐independent dilation (Table S2; Bau et al., 2005; Djousse et al., 1999; Hashimoto et al., 2001; Vlachopoulos et al., 2003). While Djousse et al. did not find acute changes in endothelial‐independent vasodilation following red wine (~23 g of alcohol; Djousse et al., 1999), Vlachopoulos et al. found a lower endothelial‐independent vasodilation following alcohol consumption (~28 g) versus the control beverage (Vlachopoulos et al., 2003). Bua et al. also reported significant decreases in endothelial‐independent vasodilation after acute alcohol consumption (60 g; Bau et al., 2005). However, following the consumption of water, de‐alcoholized red wine, vodka (~60 g of alcohol), and red wine (~60 g of alcohol), Hashimoto et al. reported endothelial‐independent dilation of 16%, 17%, 11%, and 12%, respectively, with no differences among the groups (Hashimoto et al., 2001), suggesting no effect of alcohol on endothelial‐independent vasodilation.

Three acute studies examined FMD in subjects with history of coronary artery disease and diabetes and the results are variable. In subjects with coronary artery disease, one study reported a decrease in FMD 60 min following red wine consumption (~24 g of alcohol), while the other study reported an increase in FMD 6 h following red wine consumption (~32 g of alcohol; Table 1). The changes in FMD in this population of subjects, may depend on the timing of the FMD measurements relative to alcohol consumption (Karatzi et al., 2004; Whelan et al., 2004). In subjects with type 2 diabetes, FMD and endothelial‐independent dilation was increased 3 h following the consumption of vodka (40 g of alcohol; Schaller et al., 2010). However, this study did not use a randomized design and did not include a placebo group (Schaller et al., 2010).

#### 3.5.2 Short‐term effects of alcohol on FMD

In healthy normotensive adults, four studies found no effect of short‐term moderate alcohol consumption on FMD (Andrade et al., 2009; Cuevas et al., 2000; Zilkens et al., ,^2003^, ^2005^; Table 2) or endothelial‐independent dilation (Cuevas et al., 2000; Zilkens et al., ,^2003^, ^2005^). In addition, there was no short‐term effect of alcohol consumption on FMD in subjects with arterial hypertension (Andrade et al., 2009). On the other hand, in healthy subjects receiving a high‐fat diet (Cuevas et al., 2000) or in subjects with hypercholesterolemia (Andrade et al., 2009; Coimbra et al., 2005; Cuevas et al., 2000), short‐term moderate alcohol consumption of red wine was associated with an increase in FMD. Coimbra et al. further reported that this amount of red wine consumption was also associated with an increase in endothelial‐independent dilation (Coimbra et al., 2005). In the same study, grape juice consumption was also associated with an increase in FMD (Table 2), but no change in endothelial‐independent dilation (Coimbra et al., 2005). In another short‐term study in healthy adults, Huang et al. found the daily consumption of red wine (~1 drink/day), but not beer or vodka for 3 weeks, significantly increased FMD (Huang et al., 2010). However, another study found that a 4‐week intervention in which beer consumption was decreased from heavy to mild levels did not change FMD (Zilkens et al., 2003).

#### 3.5.3 Long‐term effect of alcohol consumption on FMD

As noted above, the majority of studies including individuals with a history of lifetime, chronic or repeated alcohol consumption, included a control or abstinent/nondrinker group (Table 3). Compared to controls, there was a decrease in FMD reported in nearly all the studies that enrolled individuals with a history of chronic alcoholism (≥6 drinks/day for ≥2 years; with or without abstinence; Di Gennaro et al., 2007, 2012; Luo et al., 2017; Maiorano et al., 1999), with a history of repeated binge drinking (Goslawski et al., 2013), with a past year history of heavy drinking (>280 g/week; Oda et al., 2017, 2020), and with a lifetime history at a heavy drinking (≥46 g/day; Tanaka et al., 2016). On the other hand, while one study did not find the association of alcohol consumption over the past year with FMD (van Bussel et al., 2018), another study reported that a lifetime of alcohol consumption (1 drink/month to 2 drinks/day) was associated with an increased odds ratio for improved FMD (Suzuki et al., 2009). In men with coronary artery disease, Teragawa et al. found that compared to non‐drinkers, those who consumed ≥1 drink/week had higher FMD (Teragawa et al., 2002). In a subgroup analysis, lower amounts of daily alcohol consumption (1–20 and 21–50 g/day), but not ≥51 g/day, was associated with higher FMD (Teragawa et al., 2002).

In terms of endothelium‐independent dilation, three out of four studies in individuals with a history of chronic alcoholism (2 to 42 years of alcohol use) reported no difference in endothelial‐independent dilation versus non‐drinkers or healthy controls (Di Gennaro et al., 2007, 2012; Maiorano et al., 1999). Teragawa et al. found no changes in endothelial‐independent dilation in men with coronary artery disease and a past year history of consuming ≥1 drink/week (Teragawa et al., 2002). On the other hand, Luo et al. found 10+ years of consuming >4 drinks/day, 6–7 days/week was associated with a decreased endothelial‐independent dilation (Luo et al., 2017). Goslawski et al. reported that repeated binge drinking in young adults was associated with a significant decrease in endothelial‐independent dilation versus alcohol abstainers (Goslawski et al., 2013).

## 4 DISCUSSION

This is the first systematic review to investigate the effects of acute, short‐term, and long‐term alcohol consumption on brachial artery FMD in humans. The main findings from this systematic review include the following: (1) The acute effects of alcohol consumption have primarily been evaluated in healthy, younger individuals in which there appears to be no significant effect of light to moderate amounts of acute alcohol consumption (<3 standard drinks) on FMD, whereas a higher amount of alcohol (3–5 standard drinks) acutely and transiently decreases FMD; (2) in healthy individuals, short‐term alcohol consumption (2 to 4 weeks; ≤3 drinks/day) may not have an effect on FMD; and (3) in young and middle‐aged individuals, a history of long‐term heavy alcohol consumption (daily or binge pattern) is associated with a reduced FMD.

### 4.1 Acute effects of alcohol on FMD

Despite the variation in experimental designs, such as type of alcoholic beverage, alcohol content, and timing of FMD measurements following acute alcohol consumption, the alcohol content or dose of alcoholic beverages appears to influence the acute FMD response to alcohol: light to moderate amounts of acute alcohol consumption (<3 standard drinks) may not influence FMD, while higher amounts of alcohol (3–5 standard drinks) may acutely decrease FMD in healthy, young and middle‐aged individuals. The reduced FMD following one‐time alcohol consumption of 3–5 drinks may be associated with several factors. One of the potential mechanisms is alcohol‐induced oxidative stress (Phillips et al., 2019), which decreases bioavailability of nitric oxide and thus FMD. Polyphenols, present in red wine or fruit juices, can act as antioxidants (Scalbert et al., 2005) and thereby positively influence FMD by improving NO bioavailability. Several studies used orange/grape juices or de‐alcoholized red wine as non‐alcoholic beverages and examined the role of polyphenols on FMD following one‐time alcohol consumption, however, with conflicting findings. Some of these studies found that polyphenols may acutely increase FMD and prevent FMD reduction following alcohol (Agewall et al., 2000; Hampton et al., 2010; Hashimoto et al., 2001), while other studies suggested that polyphenols may not have effect on FMD (Boban et al., 2006; Hijmering et al., 2007; Vlachopoulos et al., 2003).

Baseline diameter of the brachial artery is an important determinant and inversely associated with FMD (Atkinson & Batterham, 2013; Silber et al., 2005). While some studies found an increase in baseline diameter of the brachial artery following one‐time alcohol consumption (Agewall et al., 2000; Hashimoto et al., 2001; Karatzi et al., 2013; Spaak et al., 2008; Vlachopoulos et al., 2003), others reported no effect of alcohol on baseline diameter (Djousse et al., 1999; Hampton et al., 2010; Hijmering et al., 2007; Muggeridge et al., 2019). Hijmering et al. found that changes in blood alcohol levels were associated with changes in FMD, but not baseline diameter of the brachial artery (Hijmering et al., 2007). Therefore, the transient changes in baseline diameter of the brachial artery, or the acute vasodilatory effect of alcohol, may not contribute to a reduction in FMD following one‐time alcohol consumption. On the other hand, allometric scaling of FMD may be useful in order to reduce the influence of baseline diameter on FMD (Atkinson & Batterham, 2013) and provide more specific information on the effect of one‐time alcohol consumption on endothelial function. FMD can reflect both the endothelial‐dependent and endothelial‐independent component of vasodilation (Maruhashi et al., 2013). To confirm whether a decrease in FMD is resulting from dysfunctional endothelium, vasodilation induced by glyceryl trinitrate, or endothelium‐independent vasodilation, should be tested. However, less than half of the acute studies in healthy individuals measured both FMD and endothelial‐independent vasodilation and findings were equivocal.

Only three studies measured blood alcohol levels (Agewall et al., 2000; Hijmering et al., 2007; Spaak et al., 2008), which are important in understanding and interpreting the effects of alcohol on physiological systems. Despite testing the same amount (concentration and volume) of alcoholic beverage consumption and normalizing to body weight, individual alcohol metabolism may influence blood alcohol level response. Future studies should incorporate the measurement of blood alcohol levels associated with the physiological parameter of interest (i.e., brachial artery FMD). This would help understand differential effects associated with high versus low amounts of alcohol consumption. Furthermore, more data is needed regarding the effects of binge levels of drinking (≥4/5 drinks for women/men in one sitting) especially in middle‐aged and older adults. This is important in light of the findings from a meta‐analysis by Grucza et al. who reported an increase in the prevalence of alcohol use and binge drinking from 2000 to 2015 for individuals ≥50 years of age (Grucza et al., 2018).

### 4.2 Short‐term effects of alcohol consumption on FMD

Based on a limited number of studies, our findings suggest that short‐term (2–4 weeks) alcohol consumption at moderate levels (2–3 drinks/day) may have no appreciable effect on FMD or endothelial‐independent vasodilation in healthy individuals. In contrast, in subjects with hyperlipidemia (Andrade et al., 2009; Coimbra et al., 2005) or in those consuming a high‐fat diet (Cuevas et al., 2000), short‐term consumption of moderate levels of alcohol may improve FMD. This improvement may be related to the effect of alcohol on increasing levels of high‐density lipoprotein (Brien et al., 2011), given that a higher level of high‐density lipoprotein is associated with a higher value of brachial artery FMD (Kuvin et al., 2003). Moderate alcohol consumption may counteract the adverse effects of high‐fat diet on FMD or may provide beneficial effects on FMD for individuals who have impaired FMD associated with abnormal lipid profiles. The effect of short‐term alcohol consumption on FMD may be influenced by type of alcoholic beverage, at least when consumed at low to moderate levels. Daily red wine consumption (~1 drink), but not daily beer or vodka consumption, may be associated with an increase in the number and function of endothelial progenitor cells and subsequent increased nitric oxide bioavailability, thus an improvement in FMD (Huang et al., 2010).

### 4.3 Long‐term effects of alcohol consumption on FMD

In the studies examining the effects of long‐term alcohol consumption, there was a variety of subject populations (e.g., individuals with a history of alcohol dependence vs. binge drinking), duration of alcohol consumption (several to many years [18–20 years]), and age range (23–71 years). Despite these variations, results from most of the included studies suggest that long‐term high levels of alcohol consumption (>3–4 drinks/day) or unhealthy drinking patterns (i.e., binge drinking, defined as ≥4 drinks for women or ≥5 drinks for men in one sitting) were associated with a decrease in brachial artery FMD. In addition, many of the studies included confounding variables (e.g., blood pressure levels, history of smoking, physical activity, and other physiologic variables, such as insulin levels) that may influence FMD or endothelial function in the statistical models. Along with the findings of acute studies which indicate that alcohol consumption of 3–5 standard drinks acutely decreases FMD, these findings suggest that the repeated exposure to high amounts of alcohol can lead to impairments in FMD, which is associated with an increased risk for future cardiovascular disease events (Inaba et al., 2010; Matsuzawa et al., 2015; Ras et al., 2013; Xu et al., 2014).

Cessation of alcohol consumption may improve the impairments in FMD associated with long‐term heavy alcohol use. In individuals with long‐term alcohol dependency, several months of abstinence (6–14 months) was associated with an improvement in FMD (Di Gennaro et al., 2012). However, despite improvements in FMD in abstinent individuals, FMD remained impaired compared to lifetime alcohol abstainers (Di Gennaro et al., 2007, 2012). In addition, these individuals had increased levels of endothelin‐1, insulin resistance, or asymmetric dimethylarginine (Di Gennaro et al., 2007, 2012). Endothelin‐1 is a strong vasoconstrictor produced by the endothelium and maintains vascular tone with nitric oxide. Insulin resistance is associated with the imbalance between endothelin‐1 and nitric oxide (Mather et al., 2013; Tabit et al., 2013), causing increased endothelin‐1 and decreased nitric oxide. Asymmetric dimethylarginine is known to inhibit the activity of endothelial nitric oxide synthase (Cooke, 2000), responsible for nitric oxide production. All these adverse changes associated with long‐term heavy alcohol use may contribute to a reduction in FMD.

Ethnic/racial differences may influence the effects of long‐term alcohol consumption on FMD. Among the five population‐based cohort cross‐sectional studies, three studies were conducted in East Asian populations (Oda et al., 2017, 2020; Tanaka et al., 2016). Results from the East Asian studies conflicted with findings from the studies conducted in Western and European Union countries (van Bussel et al., 2018; Suzuki et al., 2009). The adverse effect of heavy alcohol consumption may be more predominant in Asian individuals, possibly due to the variant genetic polymorphism of aldehyde dehydrogenase (Eng et al., 2007), influencing alcohol metabolism in the body and often leading to increased acetaldehyde levels. Acetaldehyde is a toxic substance and may induce pathophysiological changes in endothelium (Cahill & Redmond, 2012), leading to decreased FMD. The findings from the long‐term studies highlight the need to consider racial/ethnic and other characteristics in the alcohol–FMD response and more research is needed in individuals greater than 65 years of age, since only three long‐term studies enrolled subjects above this age.

### 4.4 Limitations

The overall quality of studies reviewed in this systematic review were fair. Most of the studies were conducted before 2015 and FMD measurements did not meet the current measurement guidelines (Thijssen et al., 2019). Detailed measurement and reporting of FMD requires inclusion of blood flow and shear rate/stress to ensure reproducibility of the results and to comprehensively understand the related hemodynamics related to alcohol consumption. The generalizability of our study findings may be limited to healthy, younger, and male individuals. Across all studies, women were underrepresented or not included, and for most of the studies including women, there was no information on the phase of menstrual cycle or the use of oral contraceptives. Ovarian hormones, such as estradiol, fluctuate over the course of menstrual cycle and may affect vascular function (Wenner & Stachenfeld, 2020). For example, compared to early follicular phase, FMD is higher during the late follicular phase. When comparing FMD between female subjects with different alcohol use histories, the phase of menstrual cycle or the use of oral contraceptives should be controlled to isolate the effect of alcohol on FMD. While few studies statistically considered sex as a covariate (Andrade et al., 2009; van Bussel et al., 2018; Suzuki et al., 2009), other studies may have been underpowered to examine sex differences (due to a small sample size; Agewall et al., 2000; Coimbra et al., 2005; Djousse et al., 1999; Di Gennaro et al., 2007, 2012; Goslawski et al., 2013; Hampton et al., 2010; Hijmering et al., 2007; Muggeridge et al., 2019; Spaak et al., 2008; Vlachopoulos et al., 2003). In addition, findings in most of the included studies were not presented separately for women and men.

Individuals from underrepresented minority populations are at increased risk for the development of cardiovascular disease, compared to White populations (Kurian & Cardarelli, 2007). However, most of the included studies did not provide enrollment data on the percent of underrepresented minorities. Future studies on alcohol intake and endothelial function need to include more diverse subject populations. Based on a limited number of studies, the effect of alcohol on FMD seems to be influenced by disease status. Light to moderate alcohol consumption may not change FMD in healthy individuals but may improve FMD in those with chronic diseases, such as coronary artery disease or diabetes. Future studies should also include the measurement of other cardiovascular parameters, such as blood pressure or microvascular function in conjunction with FMD to better understand the potential consequences of changes in FMD. Lastly, further investigations need to include studies of how alcohol might influence FMD across the lifespan.

### 4.5 Clinical relevance and future directions

Overall, this review found that while light to moderate alcohol consumption may not influence FMD, heavy alcohol consumption was associated with a decrease in FMD. Adults should avoid drinking three drinks or more on any single occasion to prevent FMD from decreasing acutely or permanently, which may increase future risk for cardiovascular disease. However, current evidence is based primarily on data from healthy, younger, and male individuals, with limited information on individuals from underrepresented minority populations. Alcohol consumption is projected to increase in the United States, South‐East Asia, and the Western Pacific (World Health Organization, 2018). In addition, the number of women who are current drinkers is also increasing worldwide (World Health Organization, 2018). Therefore, future studies need to include more diverse cohorts of both men and women in order to understand the effect of alcohol consumption on endothelial function across the lifespan, among diverse populations and disease status. Future studies will also need to investigate mechanisms (e.g., reduced bioavailability of nitric oxide or increased oxidant stress) underlying reduced endothelial function associated with heavy alcohol use. While studies on one‐time alcohol consumption on FMD may help understand the vascular adaptations in response to repeated consumption of alcohol, safety and ethical issues may be raised in conducting such studies, especially with heavy alcohol consumption. It appears that investigations of the effect of alcohol reduction strategies or specific abstinence interventions on FMD will be critically important to exploring the mechanisms of alcohol‐induced vascular dysfunction.

## CONFLICT OF INTEREST

None.

## AUTHOR CONTRIBUTIONS

All authors contributed to study design, selection of studies, data extraction, synthesis of study, manuscript drafting, and critical discussion.

## Supporting information



Table S1‐3
