# Supplementary material for: The effects of alcohol consumption on flow‐mediated dilation in humans: A systematic review
Source: Physiol Rep. 2021 May 27;9(10):e14872. doi: 10.14814/phy2.14872 (PMC8157766; doi:10.14814/phy2.14872)
Supplement: Supplementary file 1 — Table S1‐3 [file PHY2-9-e14872-s001.docx]

**Supplement Table 1.** Search strategies

| **Electronic medical databases** | **Mesh terms and strategies used** |
| --- | --- |
| PubMed | ("Endothelial Cells"[Mesh] OR endothelial cells[tiab] OR endothelial cell[tiab] OR endothelial[tiab] OR "Endothelium"[Mesh] OR endothelium[tiab] OR endotheliums[tiab] OR "Endothelium, Vascular"[Mesh] OR vascular endothelium[tiab]) AND ("Alcohol Drinking"[Mesh] OR alcohol[tiab] OR alcoholic beverage[tiab] OR alcoholic beverages[tiab] OR ethanol[tiab] OR "Beer"[Mesh] OR beer[tiab] OR beers[tiab] OR "Wine"[Mesh] OR wine[tiab] OR wines[tiab]) AND ("Vasodilation"[Mesh] OR vasodilation[tiab] OR vasodilatation[tiab] OR vasodilitation[tiab] OR vasodilator[tiab] OR vasodilators[tiab] OR vasodilate[tiab] OR vasodilates[tiab] OR vasodilatory[tiab] OR endothelium dependent relaxation[tiab] OR endothelial dependent relaxation[tiab] OR vasorelaxation[tiab] OR flow mediated dilation[tiab] OR flow mediated dilatation[tiab] OR FMD[tiab] OR flow mediated vasodilation[tiab] OR dilation[tiab] OR dilatation[tiab] OR dilitation[tiab] OR dilates[tiab] OR dilate[tiab] OR dilating[tiab] OR dilated[tiab]) AND English[lang] AND 1978:2020[pdat] |
| EMBASE | (endothelial cells/ or endothelial cells.tw. or endothelial cell.tw. or endothelium/ or endothelium.tw. or endotheliums.tw. or vascular endothelium/ or vascular endothelium.tw.) and (drinking behavior/ or drinking behavior.tw. or drinking behaviour.tw. or alcohol.tw. or alcoholic beverage.tw. or alcoholic beverages.tw. or ethanol.tw. or beer/ or beer.tw or beers.tw. or wine/ or wine.tw. or wines.tw.) and (vasodilation/ or vasodilation.tw. or vasodilatation.tw. or vasodilitation.tw. or vasodilator.tw. or vasodilators.tw. or vasodilate.tw. or vasodilates.tw. or vasodilatory.tw. or endothelium dependent relaxation.tw. or endothelial dependent relaxation.tw. or vasorelaxation.tw. or flow mediated dilation.tw. or flow mediated dilatation.tw. or FMD.tw. or flow mediated vasodilation.tw. or dilation.tw. or dilatation.tw.  or dilitation.tw. or dilates.tw. or dilate.tw. or dilating.tw. or dilated.tw.)  Filters: English, yr=1987-2020 |
| CINAHL | ((MH "Endothelial Cells") OR TI “endothelial cells” OR AB “endothelial cells” OR TI “endothelial cell” OR AB “endothelial cell” OR (MH "Endothelium") OR TI “endothelium” OR AB “endothelium” OR TI “endotheliums” OR AB “endotheliums” OR TI “vascular endothelium” OR AB “vascular endothelium”) AND ((MH "Alcohol Drinking") OR TI “alcohol drinking” OR AB “alcohol drinking” OR TI “alcohol” OR  AB “alcohol” OR (MH "Alcoholic Beverages") OR TI “alcoholic beverages” OR AB “alcoholic beverages” OR TI “alcoholic beverage” OR AB “alcoholic beverage” OR (MH "Ethanol") OR TI “ethanol” OR AB “ethanol” OR (MH "Wine") OR TI “wine” OR AB “wine” OR TI “wines” OR AB “wines” OR TI “beer” OR AB “beer” OR TI “beers” OR AB “beers”) AND ((MH "Vasodilation") OR TI “vasodilation” OR AB “vasodilation” OR TI “vasodilatation” OR AB “vasodilatation” OR TI “vasodilatation” OR AB “vasodilatation” OR TI “vasodilator” OR AB “vasodilator” OR TI “vasodilators” OR AB “vasodilators” OR TI “vasodilate” OR AB “vasodilate” OR TI “vasodilates” OR AB “vasodilates” OR TI “vasodilatory” OR AB “vasodilatory” OR TI “endothelium dependent relaxation” OR AB “endothelium dependent relaxation” OR TI “endothelial dependent relaxation” OR AB “endothelial dependent relaxation” OR TI “vasorelaxation” OR AB “vasorelaxation” OR TI “flow mediated dilation” OR AB “flow mediated dilation” OR TI “flow mediated dilatation” OR AB “flow mediated dilatation” OR TI “FMD” OR AB “FMD” OR TI “flow mediated vasodilation” OR AB “flow mediated vasodilation” OR TI “dilation” OR AB “dilation” OR TI “dilatation”  OR AB “dilatation” OR TI “dilitation” OR AB “dilitation” OR TI “dilates” OR AB “dilates” OR TI “dilate” OR AB “dilate” OR TI “dilating” OR “dilating” OR TI “dilated” OR AB “dilated”) AND LA English  Filters: Published Date: 19950101-20201231 |

**Supplement Table 2.** Quality assessment of included studies using the National Institute for Health and Clinical Excellence Quality Assessment Tool (N=31)

| **Authors** | **Q1^1^** | **Q2^2^** | **Q3^3^** | **Q4^4^** | **Q5^5^** | **Q6^6^** | **Q7^7^** | **Q8^8^** |
| --- | --- | --- | --- | --- | --- | --- | --- | --- |
| **Acute effects of alcohol** | | | | | | | | |
| Agewall et al. 2000 |  | X | X (limited) | X | X |  | X |  |
| Bau et al., 2005 |  | X | X (limited) | X | X |  | X |  |
| Boban et al.,2006 |  | X | X (limited) | X | X |  | X |  |
| Djousse et al., 1999 |  | X | X (limited) | X | X |  | X |  |
| Hampton et al., 2010 |  | X | X |  | X |  | X |  |
| Hashimoto et al., 2001 |  | X | X (limited) | X | X |  | X |  |
| Hijmering et al., 2007 |  | X | X | X | X |  | X |  |
| Karatzi et al., 2013 |  | X | X | X | X |  | X |  |
| Muggeridge et al., 2019 |  | X | X | X | X |  | X |  |
| Spaak et al., 2008 |  | X | X (limited) | X | X |  | X |  |
| Vlachopoulos et al., 2003 |  | X | X (limited) | X | X |  | X |  |
| Karatzi et al., 2004 |  | X | X (limited) | X | X |  | X |  |
| Whelan et al., 2004 |  | X | X | X | X |  | X |  |
| Schaller et al., 2009 |  | X | X | X | X |  | X |  |
|  |  |  |  |  |  |  |  |  |
| **Short-term effects of alcohol on FMD** | | | | | | | | |
| Andrade et al., 2009 |  | X | X | X | X |  | X | X |
| Coimbra et al., 2005 |  | X | X | X | X |  | X |  |
| Cuevas et al., 2000 |  | X | X | X | X |  | X |  |
| Huang et al., 2010 |  | X | X | X | X |  | X |  |
| Zilkens et al., 2003 |  | X | X | X | X |  | X |  |
| Zilkens et al., 2005 |  | X | X | X | X |  | X |  |
|  |  |  |  |  |  |  |  |  |
| **Cross sectional and cohort studies on FMD in individuals with a history of alcohol use** | | | | | | | | |
| Di Gennaro et al., 2007 |  | X | X | X | X |  | X | X |
| Di Gennaro et al., 2012 |  | X | X | X | X |  | X | X |
| Goslawski et al., 2013 |  | X | X | X | X |  | X | X |
| Luo et al., 2017 |  | X | X | X | X |  | X | X |
| Mairano et al. 1999 |  | X | X | X | X | X | X | X |
| Teragawa et al., 2002 |  | X | X | X | X | X | X | X |
| Oda et al., 2017 | X | X | X | X | X |  | X | X |
| Oda et al., 2020 | X | X | X | X | X |  | X | X |
| Suzuki et al.,2009 |  | X | X | X | X |  | X | X |
| Tanaka et al., 2016 | X | X | X | X | X |  | X | X |
| van Bussel et al., 2017 | X | X | X | X |  |  | X | X |

^1^ Was the case series collected in more than one center (i.e., multicenter study)?; ^2^ Is the hypothesis, aim, or objective of the study clearly described?; ^3^ Are the inclusion and exclusion criteria (case definition) clearly reported?; ^4^ Is there a clear definition of the outcomes reported?; ^5^ Were data collected prospectively?; ^6^ Is there an explicit statement that patients were recruited consecutively?; ^7^ Are the main findings of the study clearly described?; ^8^ Are outcomes stratified (e.g., by abnormal results, disease stage, patient characteristics)?

**Supplement Table 3.** Summary of brachial artery flow-mediated dilation assessment to examine endothelium-dependent vasodilation in the guidelines and included studies (N=31).

|  | **Subject Preparation** | | | **Protocol** | | | | **Technique** | |
| --- | --- | --- | --- | --- | --- | --- | --- | --- | --- |
|  | **Resting condition & position** | **Fasting, diet, medication, start time** | **Menstrual cycle** | **Cuff position** | **Occlusion pressure & duration** | **Measurement**  **timing** | **W/ GTN** | **Simultaneous live duplex ultrasound** | **Probe & Insonation** |
| **Guidelines** |  |  |  |  |  |  |  |  |  |
| Thijssen et al., 2019 | Quiet and darkened room; 10-15 min; supine | Fasted for ≥6 hr and avoid exercise for 24 hr, and caffeine or alcohol for ≥12 hr; consider medication use; standardized time of day | Standardized phase of Menstrual cycle | Distal to the imaged brachial arteries | >50 mmHg above SBP for 5 min | BL: >30 sec  Hyperemia: for ≥3 min after the cuff deflation | Yes  25 μg | Yes | ≥7.5 MHz  ≤60-70° |
| **Included study** | | | | | | | | | |
| **Acute effects of alcohol on FMD** | | | | | | | | | |
| Agewall et al. 2000 | 15 min; recumbent | 2 hours after a light standard meal; no medication; ~2 pm | NR | Forearm distal to the imaged brachial artery segment | 200 mmHg for 4.5 min | BL: NR  Hyperemia: for 2 min after the deflation | No | Yes | 10 MHz  60° angle |
| Bau et al., 2005 | Quiet and temperature-controlled room; 10 min; supine | Avoid alcohol and other psychoactive substances for 48 hr; 5 pm | - | Forearm (proximal part) | 250 mmHg for 5 min | BL: NR  Hyperemia: at 60-90 sec after the deflation | Yes  400 μg | Yes | 10 MHz  NR |
| Boban et al.,2006 | Quiet and temperature-controlled room (21°C); 15 min; supine | Fasted for ≥12 hr and avoid exercise, fruits, vegetables, dietary supplements, tea, alcohol, and caffeine or theobromine for 24 hr; 10 AM | - | Forearm | 240 mmHg for 5 min | BL: NR  Hyperemia: for 5 min after the deflation | No | Yes | 5.7- 13.3 MHz  NR |
| Djousse et al., 1999 | Quiet room; ≥ 5 min; supine | Fasted for 12 hr and avoid alcohol for a week; 8:30 am | NR | Forearm | >200 mmHg for 5 min | BL: NR  Hyperemia: from 60 sec after the deflation | Yes  400 μg | Yes | 7.5 MHz  NR |
| Hampton et al., 2010 | Supine | Fasted and avoid drinks for ~ 4hr; standard breakfast and dinner (the day before) provided; Not consumed food in high antioxidant and multivitamin, and avoid alcohol and caffeine for 5 days; 12 pm | Including subjects on contraceptive pills; ~the same phase of menstrual cycle | Forearm | 200 mmHg for 5 min | BL: 1 min  Hyperemia: for 5 min after the deflation | No | No | NR  - |
| Hashimoto et al., 2001 | Quiet and temperature-controlled (22°C to 23°C) room; 15 min; reclined | Lunch provided and then fasted; avoid alcohol for 48 hr and other beverage except water throughout the same day; 5-6 pm | - | Forearm | 250 mmHg for 5 min | BL: NR  Hyperemia: for 30 sec before and 90 sec after the deflation | Yes  300 μg | No | 7.5 MHz  - |
| Hijmering et al., 2007 | Standard and temperature-controlled room (20°C) | Fasted and avoid any drinks for ≥ 4 hr; avoid caffeine, alcohol, and smoking for 1 week; 6 pm | No information on menstrual cycle; none taking oral contraceptives | NR | NR | NR | No | Yes | 7.5-12.5 MHz  NR |
| Karatzi et al., 2013 | Quiet and temperature-controlled room (20-25°C) | Fasted for 10-12 hr and avoid alcohol, and caffeine for 20 hr; diet provided on the day before; ~8 am | - | NR | NR | NR | No | NR | NR |
| Muggeridge et al., 2019 | 10 min; supine | Fasted for ~12 hr and avoid strenuous exercise for 24 hr and caffeine for 12 hr | Post-menopausal; not on hormone replacement therapy | Upper forearm, distal to the imaged vessel | >220 mmHg for 5 min | BL: 1 min  Hyperemia: for 4 min after the deflation | No | Yes | 12 MHz  ≤60° angle |
| Spaak et al., 2008 | Quiet room; ≥10 min; supine | Light breakfast; avoid caffeine, alcohol, flavonoid-rich drink, and food from the prior afternoon; morning | NR | Forearm | 50 mmHg above SBP for 4.5 min | BL: NR  Hyperemia: for 30 sec before and 120 sec after the deflation | No | No | 7-10 MHz  - |
| Vlachopoulos et al., 2003 | Quiet and air-conditioned room; ≥10 min; supine | Fasted and avoid alcohol, caffeine, and nicotine ≥12 hr; no medication; 5-6 pm | NR | Forearm | 300 mmHg for 4.5 min | BL: NR  Hyperemia: for 30 sec before and 90 sec after the deflation | Yes  400 μg | Yes | 7.5-10.5 MHz  70° angle |
| Karatzi et al., 2004 | Quiet and temperature-controlled room (20-25°C); 10 min; supine | Fasted and avoid smoking and medication use for 12 hr; diet provided on the day before. | - | Near wrist | 250-300 mmHg for 4 min | BL: NR  Hyperemia: for 30 sec before and 90 sec after the deflation | Yes  400 μg | Yes | 7 MHz  70° angle |
| Whelan et al., 2004 | Temperature-controlled room (20°C); 10 min; supine | Light breakfast; avoid alcohol for 1 week and tea, coffee, fruit juice, and medication on the same day; ~9 am. | - | Forearm | 300 mmHg for 4.5 min | BL: NR  Hyperemia: for 30 sec before and 90 sec after the deflation | Yes  400 μg | Yes | 7.5 MHz  70° angle |
| Schaller et al., 2009 | Quiet and temperature-controlled room (22°C); | Fasted for overnight | - | Upper arm | >200 mmHg or 4.5 min | BL: 60 consecutive readings at end-diastole  Hyperemia: 180 consecutive readings at end-diastole | Yes  800 μg | No | 10 MHz  - |
| **Short-term effects of alcohol on FMD** | | | | | | | | | |
| Andrade et al., 2009 | Temperature-controlled room (20-25°C); 10 min; supine | NR; morning | NR | Forearm | 250 mmHg for 5 min | BL: NR  Hyperemia: at 1 min after the deflation | Yes  5 mg | N0 | 7.5 MHz  - |
| Coimbra et al., 2005 | Temperature-controlled room (20-25°C); 10 min; supine | NR; morning | NR | Forearm (widest part) | 250 mmHg for 5 min | BL: NR  Hyperemia: at 1 min after the deflation | Yes  5 mg | Yes | 7.5 MHz  70° angle |
| Cuevas et al., 2000 | Temperature-controlled room (22°C); 10 min; recumbent | Fasted for overnight; 8-9 am | - | Forearm | 200 mmHg for 5 min | BL: Twice  Hyperemia: at 1, 3, and 5 min after the deflation | Yes  300 μg | No | 7.5 MHz  - |
| Huang et al., 2010 | Quiet and temperature-controlled room (22-25°C) | Fasted and avoid smoking and medications for 12 hr | NR | NR | 50 mmHg above SBP for 5 min | BL: NR  Hyperemia: at 60, 100, and 12 sec after the deflation | No | Yes | 7.5 MHz  - |
| Zilkens et al., 2003 | ≥15 min; supine | Fasted and avoid beverages ≥12 hr; no smoke on the same day | - | Forearm | 200 mmHg for 5 min | BL: 1 min  Hyperemia: for 30 sec before and 4 min after the deflation | Yes  400 μg | Yes | 12 MHz  70° angle |
| Zilkens et al., 2005 | 20 min; supine | NR | - | Forearm | 200 mmHg for 5 min | BL: 2 min  Hyperemia: for 30 sec before and 3 min after the deflation | Yes  400 μg | Yes | 12 MHz  70° angle |
| **Cross sectional and cohort studies on FMD in individuals with a history of alcohol use** | | | | | | | | | |
| Di Gennaro et al., 2007 | Quiet and temperature-controlled room (22°C); 10 min; supine | Fasted overnight and avoid smoking; no medication | NR | Upper segment of the arm | 300 mmHg for 4 min | BL: NR  Hyperemia: at 60 sec, 90 sec, 180 sec, and 12 min after the deflation | Yes  400 μg | No | 7.5 MHz  - |
| Di Gennaro et al., 2012 | Quiet and temperature-controlled room (22°C); supine | Fasted for overnight and avoid smoking; no medication | NR | Upper segment of the arm | 300 mmHg for 4 min | BL: NR  Hyperemia: at 60 sec, 90 sec, 180 sec, and 12 min after the deflation | Y  400 μg | N | 7.5 MHz  - |
| Goslawski et al., 2013 | NR; supine | Fasted for overnight; morning | NR | Forearm, distal to the antecubital fossa | 60 mmHg above SBP for 5 min | BL: NR  Hyperemia: for 5 min after the deflation | Yes  400 μg | No | 11 MHz  60° angle |
| Luo et al., 2017 | Quiet and temperature-controlled room (23°C); 15 min; supine | NR | - | Forearm | 280-300 mmHg for 4 min | BL: NR  Hyperemia: at 90 sec after the deflation | Yes  400 μg | No | 10-13 MHz  - |
| Maiorano et al., 1999 | Dark and quit room; ≥10 min; supine | NR | - | Forearm, distal to the imaged vessel | >240 mmHg for 5 min | BL: NR  Hyperemia: for 20 sec before and 120 sec after the deflation | Yes  400 μg | Yes | 7 MHz  70° angle |
| Teragawa et al., 2002 | ≥10 min; supine | Vasodilatory therapies withheld for 24 hr; 6-8 am | - | Forearm | 250 mmHg for 4.5 min | BL: NR  Hyperemia: at 45-60 sec after the deflation | Yes  300 μg | Yes | 10 MHz  70° angle |
| Oda et al., 2017 | Quiet, dark, and temperature-controlled room (22-25°C); 30 min; supine | Fasted for at least 12 hr; medication allowed; ~8:30 am | - | Forearm | 50 mmHg above SBP for 5 min | BL: 30 sec  Hyperemia: for 5 min after the deflation | No | No | NR  - |
| Oda et al., 2020 | Quiet, dark, and temperature-controlled room (22-25°C); 30 min; supine | Fasted for at least 12 hr; medication allowed; ~8:30 am | Measured not during menstrual period | Forearm | 50 mmHg above SBP for 5 min | BL: 30 sec  Hyperemia: for 5 min after the deflation | No | No | NR  - |
| Suzuki et al., 2009 | Quiet, temperature-controlled room; after resting supine for 15 minutes. | Fasted for 12 hr and avoid exercise ≥4-6 hr | NR | Upper arm | 50 mmHg above SBP for 5 min | BL: NR  Hyperemia: at1 min after the deflation | No | No | 15 MHz  - |
| Tanaka et al., 2016 | NR | Fasting not required; avoid smoking and exercise for ≥ 2hr | - | Forearm | 50 mmHg above SBP for 5 min | BL: 30 sec  Hyperemia: for 20 sec before and at least 120 sec after the deflation | No | No | 10 MHz  - |
| van Bussel et al., 2017 | NR | NR | NR | Forearm | 100 mmHg above SBP for 5 min | BL: NR  Hyperemia: at 45 sec, 90 sec, 180 sec, and 300 sec after the deflation | No | Yes | 7.5 MHz  60° angle |

BL=baseline, GTN=glyceryl-trinitrate, NR= not reported, SBP=systolic blood pressure.
